# 3D-Printed Lab-on-a-Chip Diagnostic Systems-Developing a Safe-by-Design Manufacturing Approach

**DOI:** 10.3390/mi10120825

**Published:** 2019-11-28

**Authors:** Panagiotis Karayannis, Fotini Petrakli, Anastasia Gkika, Elias P. Koumoulos

**Affiliations:** Innovation in Research & Engineering Solutions (IRES), Boulevard Edmond Machtens 79/22, 1080 Brussels, Belgium; karayannisp@innovation-res.eu (P.K.); fpetrakli@innovation-res.eu (F.P.); agkika@innovation-res.eu (A.G.)

**Keywords:** 3D printing, lab-on-a-chip, Fused Filament Fabrication (FFF), safe-by-design, exposure assessment

## Abstract

The aim of this study is to provide a detailed strategy for Safe-by-Design (SbD) 3D-printed lab-on-a-chip (LOC) device manufacturing, using Fused Filament Fabrication (FFF) technology. First, the applicability of FFF in lab-on-a-chip device development is briefly discussed. Subsequently, a methodology to categorize, identify and implement SbD measures for FFF is suggested. Furthermore, the most crucial health risks involved in FFF processes are examined, placing the focus on the examination of ultrafine particle (UFP) and Volatile Organic Compound (VOC) emission hazards. Thus, a SbD scheme for lab-on-a-chip manufacturing is provided, while also taking into account process optimization for obtaining satisfactory printed LOC quality. This work can serve as a guideline for the effective application of FFF technology for lab-on-a-chip manufacturing through the safest applicable way, towards a continuous effort to support sustainable development of lab-on-a-chip devices through cost-effective means.

## 1. Introduction

### 1.1. Additive Manufacturing-FFF

Additive manufacturing (AM) is defined as the process of joining materials to make parts based on computer-generated 3D model data, usually layer upon layer, as opposed to subtractive manufacturing and formative manufacturing methodologies [1]. One of the most widely available and utilized AM techniques is known as Fused Filament Fabrication (FFF). In FFF, a solid thermoplastic filament is guided and led through a heated extrusion nozzle. Deposition requires that the process takes place at a temperature close to the filament material’s melting point. The required geometry is formed layer-by-layer through programmed extrusion of the semi-liquid material and movement of the nozzle and/or the print bed platform, which is usually also heated [2]. The most commonly used Filament materials for FFF processes are Polylactide (PLA) and Acrylonitrile butadiene styrene (ABS). Several other filament materials can be used (nylon, PETG, PC, HIPS) while filaments that include metals, nanomaterials, wood, and carbon fibres have also been manufactured, in order to generate special functional or aesthetic properties [3].

### 1.2. Lab-on-a-Chip

A lab-on-a-chip (LOC) is an automated miniaturized laboratory system, where a number of (bio)chemical processes are condensed and integrated into a single miniaturized device. The clinical diagnostics sector has been considered to be an important application of LOC systems [4]. Lab-on-a-chip devices possess significant advantages as a means for diagnostic operations, compared to commonly employed test methods in current medicine. Fast and easy diagnosis can be achieved on the precise location where it is needed (point of care – POC), requiring small quantities of materials and samples, minimizing cost of reagents and potentially improving sensitivity. These devices are superior due to their compactness, portability, modularity, reconfigurability, embedded computing, low electronic noise, limited power consumption and straightforward integration of their components [5]. They also offer reduced risk of contamination and false positives by human error concerning the carried samples [6]. Lab-on-a-chip devices are capable of supporting a wide range of processes such as sampling, routing, transport, dispensing and mixing, mostly with reduced moving or spinning components, therefore offering increased device usability and lifespan [7,8].

Microfluidics in particular, enable the manipulation and control of fluids in very small quantities within lab-on-a-chip technology. Lab-on-a-chip devices offer precise fluidic transportation via the use of electrokinetics or micropumping, efficient separation of the liquid samples and precision in the measurement of samples. Although usually the fluidic transportation pertains continuous flows, droplet based segmented flows are also favoured [9,10].

Biomedicine profits from the possibility of continuous and automatic tracking of biochemical samples and the possibility of adjusting the procedures through online feedback. The LOC devices can provide either cascade or parallel processing of samples. The advantage of parallel processing allows real-time tests of different samples with different reagents, so that the product’s effectiveness can be characterized efficiently. Lab-on-a-chip technology offers reasonable and simple maintenance of the fluidic chips. Furthermore, Lab-on-a-chip devices can be easily cleaned and sterilized with cleaning solutions such as sodium hydroxide, nitric acid, decanol, ethanol, bleach and ethylene oxide as well as through ultraviolet radiation, autoclaving and heat treatment [11].

The main applications of LOC include drug testing, HIV diagnostics [12], blood glucose monitoring and electrolytes analysis, determination of cardiac markers [13] and diagnostic operations for specific conditions such as anemia [14], diabetes [15] and cancer [16]. Lab-on-a-chip devices facilitate various biomedical tests that include specimen mixing, analysing and separating, usually consisting of cell suspensions, nucleic acids, proteins, etc. Separation methods used for lab-on-a-chip systems are smaller representations of wider approaches. The most commonly used evaluation methods are electrical and optical detection. The electrical detection methods are strongly dependent on the polar properties of the molecules of the liquid samples, while for the optical techniques labelling is required and includes chemoluminescence, fluorescence, or radioactive markers [17,18,19].

While academic publications present a highly promising outlook for the benefits that LOC devices can offer in a variety of technological sectors, widespread commercial diffusion of LOC devices to consumer products has been limited [20]. The LOC market was valued at 4.16 billion USD in 2015, and is expected to reach 9.06 billion USD by 2025 [21]. Specific research focus has been placed on enhancing accessibility and ease-of-use of lab on a chip devices, exploring the synergy with smartphones [14,22,23], or suggesting harmonized use of lab-on-a-chip with easy-to-use open source microcontrollers (e.g., Arduino [24,25]). Lab-on-a-chip development is anticipated to progress from general purpose laboratory instruments to highly personalized devices. In accordance with this movement, the utilization of widely available and inexpensively implemented technologies such as FFF as LOC manufacturing solutions can improve immediacy of lab-on-a-chip research, development and application. Establishment of a sustainable approach towards LOC manufacturing through 3D printing is critical to facilitate market diffusion and consumer acceptance, and a Safe-by-Design methodology can be pivotal in contributing to enhanced sustainability of this technological endeavour.

### 1.3. Applicability of FFF for Lab-on-a-Chip Manufacturing

The favourable characteristics of 3D printing have been employed for the development of polymer microfluidic devices. 3D printing techniques that have been implemented in research work include stereolithography (SLA) [26] and inkjet 3D printing [27]. The prospect for application of AM for microfluidic device fabrication has also attracted EC funding. The HoliFAB H2020 project aims towards innovation and industrialization of microfluidics through the use of 3D printing [28]. Τhe EC-funded M3DLOC project aims at the employment of multi-material 3D printing technologies for the large-scale fabrication of microfluidic Micro-ElectroMechanical Systems (MEMS) for lab-on-a-chip and sensing applications. The concept is based on the combination of multimaterial direct-ink-writing method and an extrusion-based 3D printing pilot line, in order to fabricate microstructured detection devices with the ability to perform all steps of chemical analysis in an automated fashion [29].

Fused filament fabrication is one of the most widely used 3D printing methods, while a multitude of materials can be used as filament feedstock. Since it is a commonly available, and easily implementable technique with relatively low-cost requirements, it has been suggested as a functional manufacturing solution for microfluidic devices. Crucially, the compatibility of FFF with lab-on-a-chip device manufacturing is challenged due to certain drawbacks of the approach that may hinder LOC device quality. These include maximum dimensional resolution, the “staircase” effect induced by the layer-by-layer operational principle, insufficient transparency, potential inconsistency between printed layers, the possibility of leaks, formation of air pockets throughout the printed structure as well as surface and intersection characteristics [30].

However, the simplicity, low cost and availability of a wide range of materials, encourages use and adaptation of the FFF technology in the field of microfluidics. Exploring the use of 3D printing for microfluidic applications, Yazdi et al. [31] have summarized a list of commercial 3D printers suitable for the fabrication of microfluidic devices, where the smaller price range of FFF 3D printers compared to instruments based on resin printing SLA and Digital Light Processing (DLP) technologies can be highlighted [31]. The higher resolution and print accuracy of these more precise techniques is accompanied by quite larger costs, which renders their application problematic for some researchers or manufacturers, while also increasing the cost of the developed device to disadvantageous levels. Additionally, in terms of safety, FFF is considered to be one of the safest additive manufacturing techniques, as no monomers or unstable chemical substances are required, no powders are employed, and biocompatible and intrinsically safe printing materials are available [32]. The main hazards involved regard emissions resulting from the printing process, high temperatures employed, and post processing procedures, and will be further elaborated upon in this study. In recent years, there has been substantial research activity in connection with identifying and quantifying potentially hazardous agent emissions from the FFF processes, as the use of FFF 3D printers is becoming more common in industry, at office workplaces, schools, libraries or for personal home use. Hazards such as ultrafine particles and VOC emissions have been confirmed and investigated. These hazards will be elaborated upon through a literature review, presented in Section 2.4*: Emissions during FFF 3D printing as presented in the literature*.

An additional benefit of FFF is the low waste potential, as the process produces practically no waste, apart from rejected/failed prints or removed support and auxiliary printing structures. Fused Filament Fabrication presents an advantage compared to other printing techniques, in that component or reagent insertion inside voids is possible, with print-pause-print methodologies [33]. Fused Filament Fabrication also presents the capability of multiple material printing, which may introduce additional capabilities for the device features and functionality.

The strengths and weaknesses of 3D printing methods regarding lab-on-a-chip manufacturing effectiveness and produced device functionality have been discussed in the literature. Zeraatkar et al. [34] performed a study in which micro mixer devices were developed with different 3D printing approaches, and then tested, aiming to highlight the limitations of each printing method. SLA, polyjet printing and FFF were evaluated, and were all determined as appropriate for microfluidic device manufacturing. The advantageous prospects of FFF in terms of low cost and the variety of filament materials are affirmed. The differences measured between nominal and printed channel depth were similar for the three fabrication methods. Fused Filament Fabrication geometries may display slightly smaller dimensions, as a result of the polymer spreading during extrusion. An interesting observation was that the roughness of FFF produced surfaces can induce a beneficial effect in terms of mixing. Fused Filament Fabrication requires the shortest channel length to achieve complete mixing, a phenomenon that was attributed to the flow being affected by macroscopic roughness elements, causing advection and increasing mixing potential [34]. Another comparative study was performed to evaluate suitability of these three printing technologies (FFF, Polyjet, DLP SLA) for manufacturing microfluidic devices, by critically comparing features, fabrication requirements and microfluidic performance of Y-junction microfluidic devices manufactured by each method. The authors report that FFF involves significant difficulties when fabricating channels smaller than 500 μm, also observing consistently smaller printed channels than nominal and higher roughness in surfaces compared to the other techniques. Mixing capabilities were high for the FFF printed device, and it was suggested that FFF can be effectively used to manufacture micromixers, or devices where mixing does not affect the output. The authors also suggest that FFF presents an advantage regarding biocompatibility, seeing that it does not involve photopolymerisation, like the other two technologies, which may involve materials with some toxic potential as well. The benefits of FFF in terms of cost, in comparison with Polyjet printing and DLP SLA were also pointed out [35]. Addressing the need for production of leak-free and impermeable microfluidic devices, Dolomite Microfluidics have developed Fluidic Factory, a specifically designed FFF 3D printer optimized for rapid prototyping of microfluidic devices using Cyclic Olefin Copolymer (COC) filament [36].

In contemporary research work, there is considerable interest to be displayed in exploiting the advantages of the FFF technique for lab on a chip manufacturing. Fused Filament Fabrication technology can be successfully implemented to produce functional lab on-a chip devices, while appropriate process parameter fine-tuning can resolve a number of the issues that are inherently present in lab-on-a-chip manufacturing through this 3D printing approach. Tothill et al. [37] designed and manufactured a microfluidic device using a commercial FFF 3D printer, using PLA and PET filaments. The authors highlight the drawbacks that common FFF filament materials present in terms of limited transparency which is a commonly needed feature in LOC devices. Four different transparent filaments were used, assessing the influence of layer height and print speed on transparency. Based on the results, it was suggested that increasing the layer height resulted in the formation of larger air pockets, reducing transparency. Increasing print speed was not found to influence transparency in a consistent manner, slightly increasing or decreasing transparency depending on the layer height of the print setup. The authors demonstrate that suitable process optimization led to the development of a functional device for performing optical colorimetric assays [37].

Morgan et al. [38] employed an inexpensive FFF 3D printer and commercially available print materials to manufacture microfluidic devices. It is reported that their methodology can resolve the issues of transparency, resolution and leakages. Several filament materials such as ABS and PET were used. The best results regarding transparency were achieved using transparent PLA with optimized print parameters. Optimization involved adjusting layer height and print speed. Reduced speeds and smaller layer height improved transparency. Optimized layer height also resulted in high dimensional fidelity of the printed structure compared to the design, with both the height and width of the channel being measured to be within 2.5% of the designed channel dimensions on average [38].

Salentijn et al. [39] demonstrate that the issue of leaks can be dealt with by modifying print parameters such as infill solidity and shell number. 100% infill density and four outlining print shells were found to display the lowest potential for leaks. With regard to the aspect of minimum achievable printable channel dimensions the authors argue that FFF may not be a viable solution when channels smaller than 100 μm are needed, but is an appropriate technique when exact fluid control through channels of this size is not needed [39]. Kadimisetty et al. [40] designed and manufactured a low cost and sensitive microfluidic immunosensor for multiple cancer protein detection, using a commercial FFF 3D printer and PLA filament. The function of the device was the detection of three prostate cancer biomarkers simultaneously in 35 min [40].

Bauer et al. [41] employed FFF technology to fabricate a diagnostic device designed to detect malaria through an ELISA (enzyme-linked immunosorbent assay) test, using ABS filament. To avoid problems of leakage that emerged, the influence of modifications on specific print settings was assessed. Increasing the infill density, as investigated in [39], was not evaluated, although being a straightforward method to diminish leakage potential, due to increased cost requirements. Print settings were optimized, so as to prevent leakages while minimally interfering with required geometry, to a 240 °C extrusion temperature, a 115 °C heating platform temperature, an extrusion speed of 60 mm/s, and a layer height of 0.16 mm. The authors suggest that such devices could be manufactured on demand in remote hospitals with 3D printing capabilities [41].

Duong et al. [42] addressed the barrier of unsuitable transparency by integrating a transparent substrate with an FFF printed chip. The authors combined the high transparency that can be offered by Poly(methyl methacrylate) (PMMA) with printed ABS components, through a method that involved printing of the required microfluidic structures with ABS filament, implementing an organic solvent bonding method for bonding between PMMA and ABS and using annealing to enhance bonding. Testing of the devices showed high mixing and emulsion production capabilities and no observed leakage. The required transparency characteristics were achieved with use of the hybrid material (White ABS and PMMA), while devices built from monolithic transparent ABS did not enable detection and identification of the specific flows [42].

### 1.4. Safe-by-Design

Safe-by-design (SbD) is a health and safety management methodology whose focal point is the identification and treatment of safety issues at the early design and R & D stages of a technological project, in contrast to approaches that aim to mitigate safety risks during manufacturing, process or use of a given technology. Through a Safe-by-design approach, the logical basis of risk management is an integral part of the design, providing continuous feedback on the selection of the most appropriate design decisions, in order to provide the safest possible end-product. Safe-by-design is based on the concept that safety can be comprehended as a functional element of a technological endeavour, like management, quality, cost and scientific objectives. The concept of SbD is established and implemented in other engineering disciplines like nuclear reactor design, workspace building design, and is considered to be highly relevant for emerging technologies such as nanotechnology [43].

Motivation to adopt a Safe-by-Design strategy can originate from the ethical mandate to evaluate safety concerns right from the onset of a technological project, or from business goals, providing cost-effective risk mitigation actions early in product development, ensuring customer satisfaction, promoting a sustainable technological development and reinforcing competitiveness. The Safe-by-design approach can be a valuable tool towards reliable lab-on-a-chip manufacturing.

**Figure 1:**
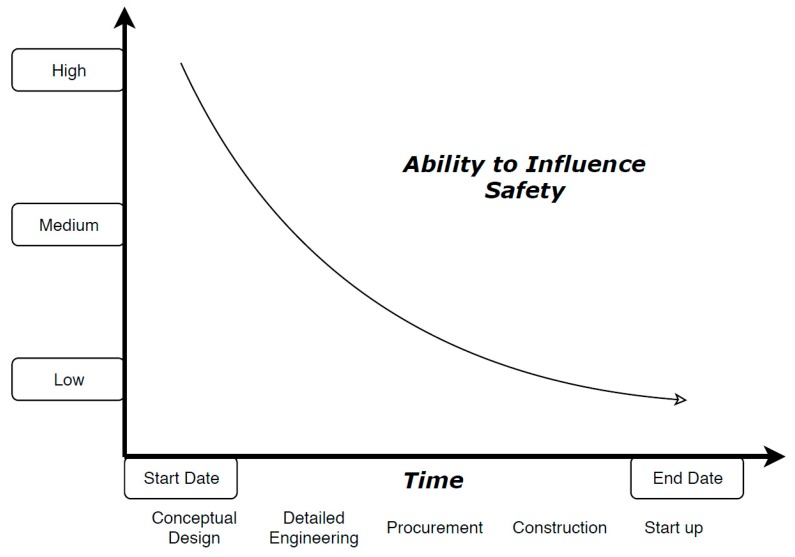
Ability to influence safety/time. Adapted from [44].

Szymberski [44] argues that the initial conception and design stages of a construction project represent a highly favourable time period to assess and potentially address safety issues, as the potential to influence safety is significantly higher compared to subsequent stages. This ability to influence safety gradually declines over time [44] (Figure 1). The logical basis of this resolution can be expanded to encompass other research and industrial disciplines as well, seeing that design phases universally present degrees of freedom towards product development not found in later stages, regardless of the nature of the project. This reasoning supports the basic functionality of Safe-by-Design strategies.

## 2. Methodology

### 2.1. Structuring a Safe-by-Design Scheme for FFF

Seeing that the manufacturing of lab-on-a-chip devices through FFF 3D printing may expose employees to risks such as UFP and VOC exposure in various extents, the establishment of safe-by-design lab-on-a-chip manufacturing should incorporate functional risk reduction measures in the context of the FFF processes, so as to ensure the maximum applicable levels of safety. The objective of this study is to describe the conception and development of a comprehensive Safe-by-Design approach to promote sustainable lab-on-a-chip device production through FFF technology.

**Figure 2:**
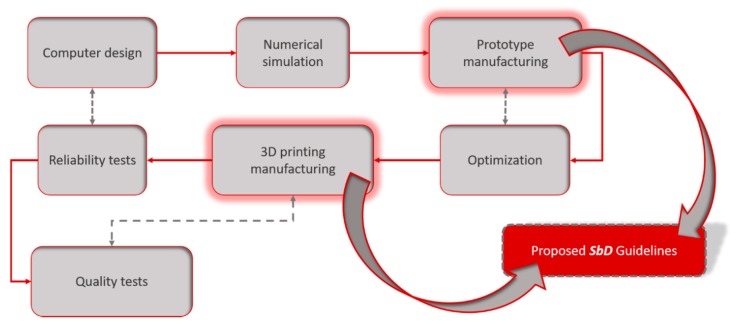
3D-printed lab-on-a-chip (LOC) development flow chart.

Development of a LOC device initiates from the design and concludes with the quality testing of the finished products. The proposed SbD approach corresponds to the manufacturing-related stages of the development procedure, which are the initial prototyping and the main manufacturing phases (Figure 2). The available knowledge about the risks involved in LOC production through FFF is adapted and restructured into guidelines that inform on adjustments that reduce risk, and a framework of process design choices that can ensure Safety-by-Design is formulated. An effective Safe-by-design scheme presupposes that a well-rounded collection of information on the safety aspects of a technology is available. This involves thorough knowledge regarding all modifiable design aspects of a process (e.g., materials, equipment functional parameters, work practices), and a risk analysis assessment and management outlook, in order to acknowledge hazards during each process stage and present the manner in which these hazards can be diminished or eliminated by adjusting specific design parameters.

**Figure 3:**
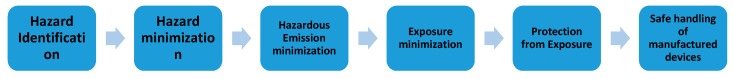
Safe-by-Design (SbD) implementation stages.

In this present SbD approach, the process of determining the most effective risk mitigation actions is organized in six stages, following the functional progression of lab-on-a-chip manufacturing: (1) Hazard Identification, (2) Hazard minimization, (3) Hazardous Emission minimization, (4) Exposure minimization, (5) Protection from exposure, (6) Safe handling of manufactured devices (Figure 3). This linear order corresponds to a hierarchy in terms of order of investigation and implementation, placing priority on earlier stages of the process. For example, adjustments that reduce the release of particles should be prioritized over mitigation actions that aim to reduce exposure after release takes place.

This safety approach is in line with the hierarchy of controls system, that prioritizes Elimination, Substitution and Engineering controls, over administrative measures and the use of Personal Protective Equipment (PPE), regarding hierarchy of application, as well as expected efficiency in reducing risk. The Hierarchy of controls can be in close connection with the Safe-by-Design mentality, as, according to this fundamental concept, the most effective controls are those that provide an extent of inherent safety. By definition, these modifications need to be taken into account from the early stages of product manufacturing.

**Figure 4:**
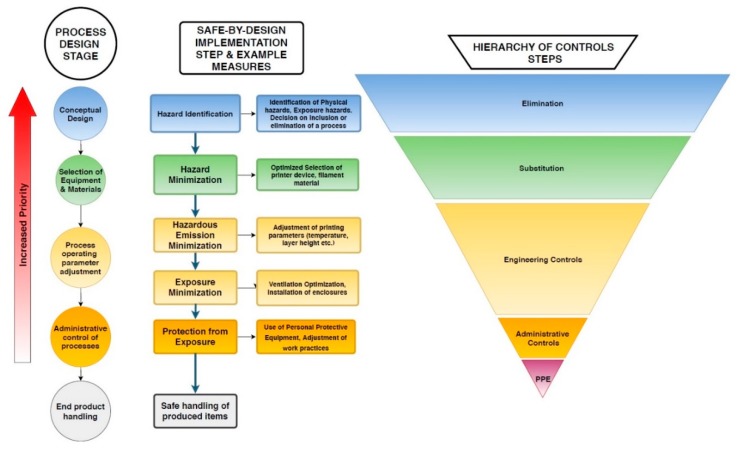
Safe by design implementation steps, in alignment with the Hierarchy of controls and process design stages.

The foundation for determining appropriate safety practices is laid through the accumulation of available information on the hazards that are expected to be encountered in the context of FFF processes. Evaluation and analysis of these findings can provide insight for confident decision-making on material selection, the adjustment of process parameters, and the establishment of effective administrative practices to facilitate safe manufacturing. Figure 4 presents an outline of the structure of the proposed SbD strategy, along with the corresponding process stage, and hierarchy of controls connection for each type of measures. In the conceptual design stages of the technology, fundamental design decisions are being made, and based on what is known about the hazards involved, specific risks can be eliminated through guided design. After the basic characteristics of the process have been determined, equipment and materials are selected. In this stage, hazardous elements can be substituted with alternatives of reduced hazard. The third stage concerns the manner in which the equipment and process operates, and how the materials are being manipulated. Insight is provided regarding process modifications, in the form of, e.g., equipment functional parameter optimization, or the installation of engineering controls (e.g., installation of ventilated enclosures) that can offer significant risk reduction. As materials, instruments, and operating conditions are fixed, the fourth stage relates to identifying optimal administrative and occupational practices, to enhance safety by protecting employees from potential exposure to hazards. The final stage is associated with the safe handling, storage and use of the produced devices.

### 2.2. Hazard Identification-the Necessity of Developing a SbD Strategy for FFF Lab-on-a-Chip Manufacturing

#### 2.2.1. Ultrafine Particle Hazards

It is acknowledged that office equipment such as laser printers and photocopiers, that utilize materials such as thermoplastic toner powder present high potential for ultrafine particle (UFP, size < 100 nm) emissions, while also possibly releasing other chemicals [45]. The functional similarity with 3D printing, as well as the fact that thermoplastics are also employed in 3D printing technologies has led to a growing need for the investigation of emissions during 3D printing processes.

Recent relevant studies have revealed potential health risks for employees involved in polymer filament 3D printer processes in case of absence of adequate controls. Emissions during desktop 3D printer processes have been reported to contain significant numbers of ultrafine particles and hazardous volatile organic compounds. The danger of acute and chronic exposure is present. Scientific work on the investigation of exposures and potential health risks is continuously expanding, while one of the main areas of focus is the standardization of release and exposure assessment procedures. In 2019, UL Chemical Safety published the ANSI/CAN/UL 2904, “Standard Method for Testing and Assessing Particle and Chemical Emissions from 3D Printers”. This standard presents characterisation and quantification protocols for particle and VOC emissions for 3D printer operation in non-industrial indoor spaces. It is to be used by stakeholders seeking to address the hazards presented within the 3D printing processes [46].

Ultrafine particles present substantial significance for the assessment of health-related risks within a workplace. UFPs can display high degrees of deposition in both the pulmonary and alveolar regions of the lung and head airways [47]. As a result, inflammatory responses may be displayed, while translocation to the brain via the olfactory nerve is also possible [48]. Contemporary scientific literature has identified a multitude of health issues that can arise as a result of exposure to ultrafine particles [49]. Furthermore, it has been demonstrated that vulnerable individuals with pre-existing respiratory and cardiovascular health issues are susceptible to displaying adverse health effects as a consequence of UFP exposure [50]. The increased biological activity of UFPs is closely associated with their increased surface area compared to larger particles [51].

#### 2.2.2. VOC Hazards

Volatile organic compounds (VOCs) are highly reactive gases that are emitted by natural sources and numerous human activities, such as smoking, vehicle operation and processes involving solvents. Inhalation of VOCs can result in short- or long-term adverse health effects being displayed [52]. Certain substances can cause irritation effects and damage to specific organs. Some VOCs are known or suspected to demonstrate carcinogenic properties.

#### 2.2.3. Fire and Explosion Hazards

Activities that generate airborne dust/particles should be investigated in terms of particle combustibility and explosion hazards. The United States Occupational Safety and Health Administration (OSHA) defines combustible dust as “a solid material composed of distinct particles or pieces, regardless of size, shape, or chemical composition, which presents a fire or deflagration hazard when suspended in air or some other oxidizing medium over a range of concentrations”. Dust particles with an effective diameter of less than 420 microns are considered to fulfil the criterion of this definition [53]. As the emission of UFP has been confirmed for FFF 3D printing processes, it is considerate to investigate combustible dust hazards in the context of FFF.

For a dust explosion to be manifested, there needs to be an airborne dispersion of combustible particles in the air or any oxidizing gaseous medium, at a particle concentration exceeding the minimum explosible dust concentration (*C*_min_) for the specific substance. A source of ignition (sparks, open flames, static electricity, hot surfaces) has to be present, and sufficient energy higher than the minimum ignition energy (MIE) must be provided. These characteristics describe the ignitability of the particles. The consequences and severity of the explosion are assessed by the maximum rate of pressure rise (*dp*/*dt*)_max_ and maximum explosion pressure (*P*_max_), indicating the explosion violence [54]. Full or partial confinement of the ignited dust cloud can lead to rapid development of pressure, flame propagation across the dust cloud and the generation of large quantities of heat and reaction products, leading to a severe explosion [55].

Aside from toxicity and adverse health symptom issues, particles in the nano-size range have been a cause for concern regarding fire and explosion hazards. Parameters that can influence the explosion hazard of air suspended powders can be particle shape, particle size distribution (PSD), degree of agglomeration, dust concentration in the cloud and degree of turbulence suspension [56]. The emission of ultrafine particles in the context of FFF 3D printing processes has been acknowledged, however, to our best of knowledge, no systematic study of the explosive hazard of these specific emissions has been undertaken as of yet. Therefore, critical information such as the minimum explosible dust concentration is unavailable. The surpassing of the MIE is highly unlikely for well ventilated spaces; however, it may be a possibility in cases of multiple printer operation in confined, poorly ventilated spaces.

Ignition ease, severity and propagation is considered to increase as a result of smaller particle size [57]. Smaller particles will be gasified quickly as a result of increased surface area, and gasification of the combustible particles is highly important for flame propagation [54]. Based on this, it is to be expected that nanoparticles would display higher explosion propensity and severity compared to larger particles. Physicochemical properties of particles can change significantly when size is reduced to <100 nm, so their explosion hazard properties may not be easily and clearly determined. Agglomeration may display an important role, as it is a naturally expected property of nanoparticles, and is enhanced further by the effect of the continuous random Brownian motion. Explosion related properties of agglomerated and aggregated particles cannot be predicted with total accuracy, however a decrease in the effective surface area is to be expected. Therefore, displayed properties may be similar to those of larger particles than the primary nanoparticles.

The Health and Safety Executive (HSE) developed a specially designed test apparatus, to measure the explosion characteristics of nanosized powders, and investigated materials such aluminium, iron, copper, zinc nanopowders and carbon nanotubes, nanofibers and nanopowder as well as corresponding material micron powders. Results showed similar explosion violence (maximum explosion pressure, rates of pressure rise and KSt) of nanopowders and micron-scale powders. Minimum ignition energies of iron and zinc nanopowders were assessed to be lower than the equivalent material at micron-scale, suggesting high susceptibility to ignition. Carbon nanotube materials were less susceptible to ignition, displaying minimum ignition energies greater than 1000 mJ. Regarding aluminium nanopowders, minimum ignition energies of aluminium nanopowders were comparable with micron-scale aluminium at very low (<1 mJ) values, indicating great sensitivity to ignition [58]. Importantly, this study notes that to pose a fire risk, nanopowders need to be present in large quantities of grams, even kilograms. Ιt should be acknowledged that there should be accumulation of high amounts of airborne dust for the explosion hazard to be of substantial concern. Eckhoff [59] presents valid reasoning that nanoscale particles may not display extreme sensitivity to explosions, as expected from extrapolation of the microscale trend, highlighting inter-particle cohesion forces, limited dispersibility, and high coagulation rate as determining factors. It is suggested that for most organic materials, reducing particle size below 10 μm will no longer influence the explosion violence/rate, as the rate controlling process for flame propagation is the combustion of pyrolysis gases/volatiles. However, the propensity of some metal nm-powders to display lower MIE values is highlighted [59].

### 2.3. Printer Operation Hazards

**Figure 5:**
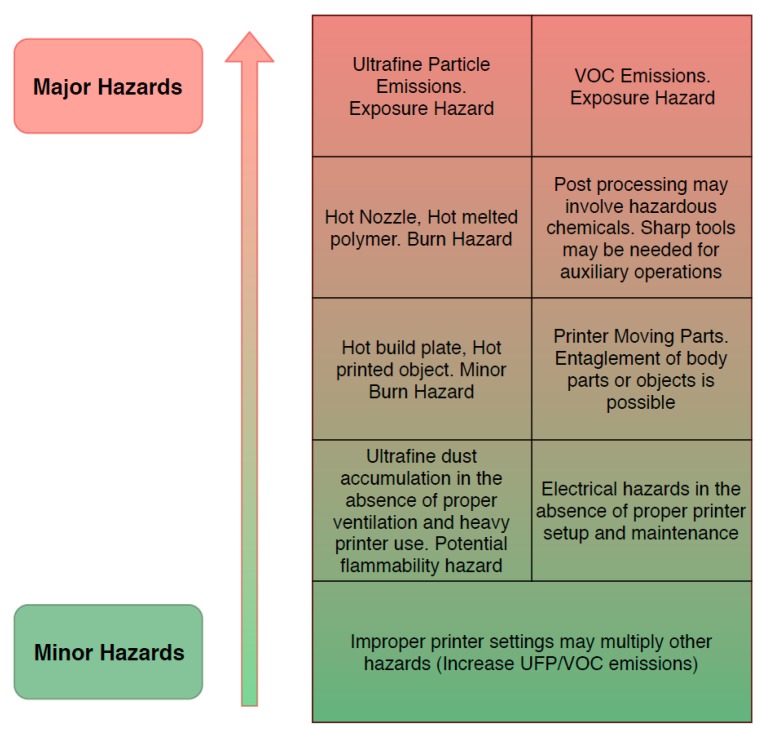
Hazard identification in Fused Filament Fabrication (FFF) 3D printing process.

The FFF process can present several other hazards of various types. Additionally, print preparation and post processing procedures may introduce distinct hazards. Figure 5 presents a list of the hazards exhibited in FFF processes, prioritized based on their severity.

#### 2.3.1. Electrical Hazards

High power voltage can present the risk of injury due to electrical shock. Exposed printer parts employ low voltage (no higher than 12 or 24 V). Higher voltage can generally be encountered when removing equipment covers. In CE marked printers the power supply will be protected against short-circuit, overload, over-voltage and over-temperature, according to regulations. Some commercial FFF 3D printers are supplied in an assembly kit. This presents increased probability of improper setup and function, raising electrical risk levels as a result of coming into contact with live parts.

#### 2.3.2. Ergonomic Hazards

Moving printer parts may display speed and movement strength considerable enough to cause minor injuries. Entanglement and trapping of hair, clothing, jewellery and body parts due to with moving printer parts can also occur. Printer lifting and transportation may present hazards of musculoskeletal injury, due to heavy printer weight.

#### 2.3.3. High Temperature Hazards

During function, some surfaces of the printer are very hot. Print bed temperatures can extend to 120 °C, and typical print nozzle temperatures are 180–250 °C. Contact with these parts, or interfering with the melted material while it is extruded without any protective measures may induce skin burns and scalds. The printed object is very hot immediately after print completion, and can also present the potential for inducing burn damage if removed promptly.

#### 2.3.4. Hazards Related to Use of Auxiliary Tools/Processes

Print removal from bed and printed object post processing may involve sharp tools, presenting a cutting hazard. Sharp tools may be needed to remove support structures as well. Depending on the application, printed objects may display sharp edges. Additionally, Sprays or glues to enhance printing object adhesion to print bed are often used. The frequent use of sprays in inadequately ventilated spaces may deteriorate air quality, while the sprays may have irritation effects [60]. Some post processing methods require the use of potentially dangerous substances (e.g., caustic baths to remove supports, or polishing procedures using agents such as acetone or chloroform).

#### 2.3.5. Noise Hazards

Most 3D printer devices are not able to produce significant levels of noise. Operation of multiple 3D printers may result in increased noise levels, leading to potential employee frustration and reduced concentration, although not expected to reach hazardously high sound levels. Enclosures can mitigate any noise hazards.

#### 2.3.6. Software Hazards

Hazards due to software issues may also manifest. Interconnectibility with smartphones, and computers is common practice in contemporary 3D printing and offer significant benefits but may increase probability of incompatibility and malfunction if employed inappropriately. Also, modifications in firmware, that may be used to maximize printer efficiency can lead to unstable device operation. Incompetent slicing software operation may lead to improper print settings (e.g., using excessively high nozzle temperature) and print malfunctions, which can have undesired consequences and new hazards may emerge (e.g., the need to unclog the nozzle employing high temperature, sharp tools or with the use of chemicals).

### 2.4. Emissions during FFF 3D Printing as Presented in the Literature

The main health hazard that is present in FFF operations is exposure to the emissions resulting from printer function. In the following paragraphs, a summary of the representative research work studying emissions in connection with the use of 3D printers is presented. The focal point of the literature examination is the identification of risk reducing measures through the interpretation of the study findings. These measures are then incorporated in the comprehensive SbD strategy.

As a logical basis for the emission investigation, it is important to note that the potential for emission of hazardous agents as a result of polymer thermal processing in general has been confirmed. Guillemot et al. [61] investigated the thermal behaviour of thermoplastics and study results have shown that when polymers are subjected to temperatures from 150 °C to 450 °C for producing plastic products through processes such as injection moulding and extrusion, this processing can lead to emissions of VOCs in laboratories and industrial workplaces [61]. Such emissions within the context of thermal processing processes have also been reported by Unwin et al. [62], even in low temperatures (200–250 °C). When proper control measures, such as adequate ventilation or temperature control are applied, the concentrations were found to be within acceptable levels [62].

#### 2.4.1. Quantifying FFF Emissions

In one of the earliest endeavours to assess 3D printer emissions in 2013, Stephens et al. [63] studied the emission potential of two commercial 3D printers inside a small office space. The authors conducted measurements of size-resolved and total ultrafine particle concentrations during printer operation. They investigated the most common filament materials, reporting emission rates of ≈2.0 × 10^10^ #/min for PLA feedstock and ≈1.9 × 10^11^ #/min for ABS feedstock (#–corresponds to number of particles). Detected particles were reported to be smaller than 150 nm. No elevations in particle concentrations for particles larger than 116 nm in size were observed. It was also demonstrated that peak emission rates from the PLA-based printers involved particles in the 48–65 nm size range while ABS-based printer peak emission rates occurred in the ≈15–49 nm size range. The authors acknowledge that these devices are commonly supplied without any exhaust ventilation or filtration accessories, and point out that adequate ventilation and air filtration systems should be in place to ensure safe use [63].

Zhou et al. [64] investigated the particle emission rates of commercial desktop 3D printers using several types of ABS as filaments, inside a Class 10,000 clean room. Particle concentration measurements were conducted at three different positions within the clean room, to assess the influence of the relative distance from the printer. It is important to note that ultrafine particle emissions were not assessed, and only emitted particles in the 0.25–32 μm size range were measured. The highest concentration levels were observed for the smallest observable particles, in the size range of 0.25–0.28 μm, and particle concentration reached a maximum of 5 × 10^4^ #/L. The function of two printers resulted in higher maximum particle concentration (≈7 × 10^4^ #/L). For particles larger than 0.375 μm, very low concentrations were reported. For measurement positions further from the printer, higher concentrations were observed. The authors suggested that this observation can be attributed to the growth of ultrafine particles emitted from the printers. Coagulation may take place as particles transport away from the printers. After printing completion, the ventilation system was turned on and rapid removal of the particles was achieved [64].

Kim et al. [65] investigated emissions originating from two different printers using ABS and two different types of PLA filaments (PLA1, PLA2). Compared to outdoor conditions and measurements before and after print operation, higher number concentrations during printing were observed for all filament materials. The authors reported significantly higher particle concentrations (33−38 times higher) when ABS was used. Most emitted particles were nanosized for ABS (96% of particles <100 nm) and PLA1 (98% of particles <100 nm), but not for PLA2 (12% of particles <100 nm). The highest peaks in PNC were observed at the beginning of printing. For PLA filaments, VOCs such as toluene and ethylbenzene were detected. For one of the PLA materials (PLA2), a rise in formaldehyde concentrations (5.2 times higher than the outdoor concentration) was also detected [65]. Toluene [66] and ethylbenzene [67] are considered health hazards and formaldehyde has been described as a “potential occupational carcinogen” and is considered an acute toxic substance [68].

Azimi et al. [69] quantified emissions of UFPs and VOCs for five commercial filament extrusion desktop 3D printers using up to nine different filaments: ABS, PLA, high impact polystyrene (HIPS), semitransparent nylon, laybrick, laywood, transparent polycarbonate, a semitransparent nylon-based plasticized copolyamide thermoplastic elastomer (PCTPE), and a transparent polyester resin filament called TGlase. The influence of bed temperature (ranging from room temperature to 110 °C, according to print requirements) and nozzle temperature (varying from 190 to 270 °C) was investigated. It was found that of all the filament materials tested, ABS presented the highest emission rates while PLA presented the lowest rates. All other materials displayed emission rates in between. Regarding VOCs, PLA filaments mainly emitted lactide with emission rates in the range of ~4 to ~5 μg/min. Lactide can induce severe eye irritation, and skin corrosion and irritation [70]. Printers with the highest bed temperature had the highest particle emission rates. Extruder temperature was not found to greatly influence particle emission rates. Higher extruder temperatures were found to result in significantly higher emissions only for midrange bed temperatures (60–65 °C). Between different print shapes that require similar print time, the shape geometry was not found to change the emission rates considerably. A partial enclosure resulted in a ~35% reduction in the median emission rate. Based on the results produced, the authors calculated a worst-case exposure profile for a hypothetical office space with typical office ventilation parameters. It was found that a printer operating under the assumed conditions would lead to hazardous VOC concentrations and elevated concentrations for UFP, compared to typical office spaces [69].

Deng et al. [71] examined particle emissions of 3D printing processes using ABS and PLA filaments, during loading, heating, printing, and unloading process steps. It was demonstrated that particle emissions can be attributed to the heating process rather than the printing process. The authors demonstrate that particle emissions can increase by orders of magnitude for both types of filaments when nozzle temperature increased from the lower to upper end (180–200 °C for PLA). Further temperature increase to 220 °C, reaching PLA’s decomposition temperature, resulted in particle emissions being increased substantially. For temperatures above 200 °C, emissions were recorded to be in the range of 20,000–40,000 #/cm^3^, displaying significant fluctuation during the printing process. The influence of feed rate was also investigated. Utilization of a middle range feed rate (60 mm/s) was found to result in higher emissions compared to lower (30 mm/s) or higher range (90 mm/s) feed rates. When working with PLA, the authors suggest adjusting nozzle temperature to 180 °C, combined with a fast feed rate (90 mm/s). In terms of product quality, the PLA filament was reported to be highly tolerant to temperature and feed rate modifications. Seeing that the heating stage was determined as the prevalent contributing mechanism for the emissions, the authors tested externally heating the extruder and platform to the desired temperatures before filament loading. Compared to conventional pre-heating, this reduced particle emissions by 75% for the ABS filament [71].

Infill characteristics have been reported to affect emissions. This is a parameter that is highly likely to be adjusted in 3D printing processes according to the application requirements, as it heavily influences filament material used, print time, printed object weight and mechanical properties such as Young’s modulus [72]. Cheng et al. [73] used ABS filaments to print several object shapes and investigate the emission impact of modifications on infill density, pattern and height. The authors report that the widely used hexagonal infill pattern results in comparatively high emissions and suggest using the linear pattern as a solution that offers a combination of satisfactory results in terms of print time and emission potential, while being easily available in slicing software. Interestingly, this research work highlights several emission observations. A critical one is that peak concentrations (≈100 fold higher than the emissions during the rest of the print time (≈ 2.6 × 10^7^ #/m^3^) were found to occur when the design of the printed object required flat top solid layers after infill to be printed. The effect defined as “bridging” (printing from one contact point to another without supporting material being present underneath), was determined to be the main reason that peak emissions occurred. The peak emission magnitude was also demonstrated to increase, as the infill height increased. In terms of infill density, an increase of the density from 10, 20, 30% (the density range expected for most mechanically non-demanding prints) results in the peak concentrations being significantly reduced by 33% and 78% respectively. The authors tested modifying the feed rate on the layer that resulted in the manifestation of the bridging effect to reduce emissions, achieving 47% and 65% reduction of the peak emissions as the feed rate of this layer was reduced from 60 mm/s, to 45 mm/s and 30 mm/s, respectively. Combination of the optimal density and feed rate settings resulted in a 96% reduction of the peak emission value. A suggestion is also made to schedule operation of used particle filtering devices according to the expected peak emission time frames, in order to reduce energy requirements and cost [73].

Yi et al. [74] investigated the determinant factors that control particulate emissions from 3D printers and also performed characterisation of the emitted particles. This study demonstrated that the utilization of ABS resulted in larger emitted particles than PLA (99% of particles <100 nm for PLA), and alveolar deposition was calculated to be threefold higher for PLA than ABS. Furthermore, a loose-fitting cover was found to be able to reduce total particle number emissions by a factor of 2. Differences in emissions due to different filament color for the same filament material were connected with the unique additives used for the colouring purposes. An interesting observation was that an upsurge in emissions can be brought about as a result of a printer nozzle jam. This was attributed to the continuous heating of the nozzle during this episode, while inefficient heat transfer to the filament takes place because of the jam [74].

Zhang et al. [75] conducted a quantitative exposure assessment for six commercial FFF 3D printers during operation in different environments, in order to investigate particle emissions. The investigation involved three types of widely used filament materials (ABS, PLA, nylon). This study indicated several parameters that may influence emission potential, including extruder temperature, filament brand (due to differences in trace components) and filament colour and build plate temperature, albeit of reduced impact. It was suggested that emissions from the material used could be investigated and studied as a function of temperature. The authors report that most particles were detected to be smaller than 100 nm, instantaneous particle number concentration can reach the range of 10^6^ #/cm^3^ and particle number emission rates can reach 10^11^ #/min. It was pointed out that both public and personal use of 3D printers may result in the emergence of health risks as a consequence of the high exposure potential, especially in cases of use by vulnerable individuals [75].

Simon et al. [76] investigated the energy consumption and particle emission potential of FFF in connection with modifications in print settings. ABS filament material and a commercial 3D printer were used, while particle emissions were monitored with appropriate equipment in a fume hood inside an ISO 14644-1 class 3 cleanroom. A 10–420 nm particle emission spike of ~210,000#/cm^3^ when the target extruder (230 °C) and print bed (110 °C) temperatures were approximated was observed. Smaller, although substantial spikes were caused by pausing of the printing process. Interestingly, the intensity of the peaks was not significantly affected by pause duration. Increase of material flow also led to reduction of particle emissions. Higher speeds and increased material flow decrease the filament residence time in the nozzle, which is where the lower emissions are attributed to. For particles larger than 420 nm, no statistical difference in PNC was observed. Particle size distribution of emitted particles showed that most particles were less than 25 nm in diameter. Reduction of particle emissions was achieved by clearing the nozzle of residual filament, by clearing its interior with a wire and heating it to 260 °C for 24 h. The authors suggest cleaning the nozzle after each run and using higher material flow rate as emission reduction methods, and employing higher print speed to reduce energy requirements, if applicable [76].

#### 2.4.2. Assessment and Characterisation of the Emissions

In order to estimate the hazards involved, an assessment of the properties of the emitted particles is important. Several studies have examined the specific characteristics of the emitted particles in FFF 3D printing processes. Steinle [77] measured and characterized emissions of a desktop FDM printer using PLA and ABS filaments, in two different rooms (well- and poorly- ventilated). It was found that emissions of UFPs and VOCs were relatively high for both materials in the poorly ventilated room case. Emissions consisted mainly of volatile droplets, while detecting soot-like particles (C-containing agglomerates) as well. It was demonstrated that for PLA filaments, methyl methacrylate (MMA, 37% of TVOC) was determined as the prevalent compound of the total VOC emitted. It reached a peak concentration of 21 μg/m^3^ in the inadequately ventilated room, and was detectable many hours after printing. MMA is considered a respiratory and dermal sensitizer and respiratory irritant [78]. The detection of Traces of fluoranthene and pyrene, as well as Fe and Zn, was also reported. The hypothesis was made that iron particles may be present in the filaments as impurities, or be emitted by abrasion from the printer’s mechanical parts. Contamination from metal handling tools used during the process was also stated as a possibility. Other remarks were that prolonged use of printers can lead to higher emission rates, while lighter objects and objects where print duration was shorter, displayed comparatively higher relative emission rates [77].

Wojtyła et al. [79] investigated thermal decomposition of commercially available thermoplastic filaments: (ABS, PLA, PET and nylon) using TGA for identifying thermal patterns and GC analysis of emitted VOC. The authors report that the temperatures used may be relatively low, compared to the total decomposition temperatures of these materials, but they are high enough for causing partial decomposition of polymers with emission of volatile organic compounds. For PLA, methyl methacrylate has been detected as the predominant compound (44% of total emitted VOCs) [79]. In a recent study, Wojtyła et al. [80] suggest a universal method to evaluate and compare filaments in terms of VOC emission potential independent from the influence of printer parameters and printing settings (ex vivo approach). Different types of filaments were heated to temperatures reaching the upper printing limit recommended by the manufacturer of each filament and gas from above the sample was analysed using gas chromatography. Highest VOC concentrations were observed for styrene, acrylonitrile and ethylbenzene, for ABS and HIPS filaments. Nylon was found to emit caprolactam, which is considered an irritant [81]. PLA emitted lactide and lactic acid. The impact of temperature on VOC emission was also observed, although filament type was found to be a prevalent determining factor over temperature. Color was found to display an important role as well, as it signifies the inclusion of certain additives. Carbon-fibre containing filaments were reported to be low emitters compared to ABS and HIPS, and this reduction was attributed to the presence of the CFs as thermal stability enhancing factors; however, some hazardous VOCs such as cumene [82] and acetonitrile were identified. The authors note that the type of emissions may be connected not only to thermal degradation of the polymer, but to the decomposition of chemical additives (dyes, fillers plasticizers, flame retardant) [80].

Stefaniak et al. [83] evaluated atmospheres in four workplaces utilizing 22 FFF desktop 3D printers. Airborne particle diameter, number concentration and total volatile organic compound concentrations were measured using real-time instruments. Particle emission rates ranged from 10^9^ to 10^11^ #/min and organic chemical concentration in workplace air displayed great variation. Influencing parameters were, among others, instrument design, filament material and build settings. The types of VOCs and their concentrations varied among facilities. All personal VOC levels were well below applicable National Institute for Occupational Safety and Health (NIOSH) Recommended Exposure Limit (REL) values [83].

Zontek et al. [84] characterized particle emissions originating from two commonly employed 3D printers using PLA and ABS, measuring particle concentrations in different positions inside two differently ventilated (well- and poorly-ventilated) process workspaces. Consistent results with other studies were presented in terms of the influence of temperature, reporting that higher printing temperatures resulted in higher number particle concentrations. Reversely to [64], measurements inside the well-ventilated workspace indicate that particle concentration fell off significantly while measurement distance from the printer increased. Particle concentration reached 3000 #/cm^3^. Approximately 3/4 of the room (10 m × 10 m × 6 m, 20 AC [Air Changes]/h) maintained particle concentrations approximate to background level. For the room with poor ventilation (3 m × 9 m × 6 m, 1.8 AC/h), particle concentration increased to 10^4^ #/cm^3^ near the breathing zone of the employees, close to the printer, while continuous function of the printer resulted in the surrounding room concentration reaching approximate levels. The particles emitted from PLA and ABS were found to be composed of individual and aggregated particles containing carbon, oxygen and various metallic elements (Na, Al, Cu and Mg for PLA filaments). It was assessed that carbon agglomerates, along with some of the metals identified, suggest an aerosol capable of generating reactive oxygen species. Examining the inconsistency regarding the magnitude of the emissions compared to Stephens et al. [63], it was deduced that the higher emissions presented in that study were a consequence of printing smaller artifacts with shorter printing times. The authors point out that further research work should focus on providing ventilation recommendations, and determining suitable printer locations with respect to occupied locations. They recommend that printers without enclosures should be restricted for use in large, highly ventilated spaces, and highlight that special care should be given when asthmatic or individuals with respiratory issues are assigned to such processes, through the implementation of local exhaust ventilation. Maintaining the printer enclosure under slight negative pressure with respect to the surroundings is also mentioned as a control strategy [84].

Floyd et at. [85] characterized aerosols and VOC emissions generated from various filaments used with a low-cost 3D printer in an environmental testing chamber. Eight filament types with diameters of 1.75 mm were used, namely ABS, PLA, PVA, HIPS, PCABS, nylon, bronze- PLA, and PET, while the nozzle and baseplate temperatures were fixed at 210 °C and 70 °C, respectively, to reduce the number of experimental variables. The authors report that there is potential for exposure to high concentrations of nanoparticles for users of low-cost 3-D printers. The ABS-based filament (ABS and PCABS), as well as the bronze-infilled PLA, and the PVA filaments resulted in the highest particle emissions. Regarding both concentration and size distribution, large numbers of particles were emitted at peak magnitudes of 10^11^ #/min, with a modal size of less than 100 nm, thus presenting higher probability of penetrating to the alveolar region of the respiratory tract. Rod-shaped fragments were also observed. Particles of this shape can pose higher pulmonary risks as they can be trapped in the small airways. PLA-based filaments emitted acrylic acid dimer at a rate of 6–11 μg/min. The study was conducted in an environmental chamber and therefore should not directly be compared with exposure limits [85].

Mendes et al. [86] evaluated the emission potential of a low-end 3D printer using PLA and ABS. It was found that PLA printing presented negligible UFP concentrations, when the recommended settings were used (*T*_extruder_ = 200 °C). However, particle concentrations were significantly higher (more than 3 × 10^3^ #/cm^3^), when higher than recommended temperature was employed (T_extruder_ = 230 °C). For ABS, increasing the nozzle temperature from 238 °C to 250 °C was found to increase the particle emission rate ten times on average. According to this study, traceable amounts of formaldehyde, acetaldehyde, and acetone were emitted during printing with ABS and PLA, but the amounts of VOCs emitted were negligible, and were not considered sufficient to pose health risks. Nevertheless, this is the only study that reports significant emission rates of nanoparticles below the 10 nm range (1–3 nm). Another interesting finding was that malfunction of the printing process can lead to higher emissions. Malfunction episodes were observed as a result of not using utility products to increase the adhesion of the printed object to the bed, and involved transport of the position of the object and sticking of the filament to the nozzle. This study suggests comparison of exposure measurements with the nano reference value (NRV) of 4 × 10^4^ #/cm^3^ (8-h time-weighted average [TWA]) for bio-persistent nanomaterials of density lower than 6000 kg/m^3^ [87], used as an indicative exposure limit. They report that emission concentrations from PLA are below this threshold, but this limit was reached and exceeded slightly in the case of ABS filaments. The authors suggest that exposure control measures should be implemented when working with low end printers, and also recommend caution when operating several printers simultaneously, as may be expected in many occupational printing applications [86].

The Health and Safety Executive (HSE) [88] set up an interdisciplinary working group in order to investigate emissions from FFF 3D printers through laboratory experiments. They tested PLA, ABS, High Impact Polystyrene (HIPS) and NinjaFlex^®^ (thermoplastic polyurethane) as filament materials in three 3D printer models, according to printer-material compatibility, reporting that all of the 3D FFF printers emitted sub-micrometre particles regardless of the filament used. It was also observed that increase of the nozzle temperature resulted in the average particle size being decreased and the emission rate being increased. For PLA filaments, using 220 °C as a nozzle temperature resulted in emission rates in the range of 3.26–8.08 × 10^9^ #/min with average particle sizes in the range of 45–69 nm, while the higher 240 °C temperature was found to result in emission rates in the range of 4.64–5.72 × 10^10^ #/min with average particle sizes in the range of 32–34 nm. These results are comparable with [63]. PLA filaments were found to display lower emissions than ABS in this study as well. However, comparing their results with He et al. [89], where traditional printers utilized in office settings were evaluated in terms of emissions, the authors point out that the lowest emission rate for PLA filament was two orders of magnitude greater than the lowest measured for office printers. Pyrolysis testing revealed that PLA mainly produced lactide as VOC emissions, in accordance with [69]. It was pointed out that more experiments need to be conducted, in environments of real workplace conditions, as opposed to controlled laboratory studies, in order to understand the findings more clearly, while also assessing exposure to 3D printer emissions, for long-term and regular users of such equipment [88].

Bharti and Singh [90] studied the exposure potential of 3D printer use in libraries, where ventilation is not designed for 3D printer use, thus raising valid concerns about increased UFP count, inefficient removal, and the possible exposure for numerous individuals. The authors studied the emission characteristics resulting from the function of 5 non-enclosed 3D printers using PLA filament and default manufacturer print settings, within a ~225 ft^2^ room. The simultaneous operation of three, four and five printers was examined. UFP particle concentrations were found to be much higher in the printing room compared to other sites within the library (26–36 times higher than the control locations and 17 times higher that concentrations in the adjacent room). Furthermore, the average UFPs concentration in the 3D printing room increased substantially when five printers were operational compared to function of four or three printers, reaching 86.995 #/cm^3^. It is interesting to note that this operation setup results in emission levels that exceed the relevant proposed exposure limit that corresponds to biopersistent nanoparticles (40,000 #/cm^3^), and was also suggested by [86], to a substantial degree. The average UFP number concentration for three printers operating (39,400#/cm^3^) approximated this threshold, while operation of four printers (52,760 #/cm^3^) exceeded this exposure limit as well [90].

In another study, the emission potential from desktop as well as industrial scale FFF 3D printers was investigated by du Preez et al. [91]. This work also examined the post processing polishing procedures of the printed parts in terms of UFP and VOC exposure. It was demonstrated that the opening of the industrial-scale 3-D printer doors after printing did not result in significant particle number concentration elevation, but brought about a short-term increase in TVOC concentration. The authors attributed this phenomenon to the time that had passed from build completion to the door opening (several hours), resulting in particle decay via settling and/or adherence to the interior walls of the build chamber. For the desktop printers, cover removal resulted in increased particle number concentrations at the interface of the printer and room air. Filament type and colour was found to be a determining factor of the emission magnitude, exceeding 200,000 #/cm^3^ for black ABS, while lower particle count was observed for red PLA (50,000 #/cm^3^) and other materials (green PLA, blue ABS and light blue PLA < 50,000 #/cm^3^). TVOC concentration was found to increase during specific stages of the acetone-based polishing processes for the ABS object polishing procedure, and, to a lesser magnitude the chloroform-based PLA object polishing procedure [91].

In a recent study, Youn et al. [92] performed an investigation of FFF emissions, measuring and sampling airborne nanoparticles and hazardous air pollutants at a 3D printing centre employing five FFF 3D printers utilizing PLA filament feedstock. The authors tested sequential and simultaneous use of the five printers, and also estimated emitted nanoparticle deposition in sectors of the respiratory system in the event of human exposure. It was reported that the nanoparticles emitted exhibited a bimodal size distribution, with dominant peaks at 10 nm (expressed primarily during the initiation of printer operation) and 88 nm (expressed after a short time interval). These particles were of approximately spherical shape, their mean size was found to be 19.85 nm, and agglomerated to form larger particles approximately 100 nm in size. The FFF process was also found to result in 14 Hazardous Air Pollutants (HAP) species generation. Concentrations were generally low, but seeing that these substances contained class 1 confirmed human carcinogens (benzene and trichloroethylene) and class 2, probable or possible carcinogens (acrylonitrile, methylene chloride, chloroform, tetrachloroethylene, styrene) as well as agents affecting to the Central Nervous system, and several irritants, prolonged exposure may present hazard. Regarding particle deposition on the human respiratory system, it was assessed that larger numbers of nanoparticles are deposited in the lower respiratory tract compared to the upper respiratory tract. According to these results, the nanoparticles deposit in the pulmonary region which consists primarily of alveolar airways [92].

Rao et al. [93] used nanofiber-based air filters to collect PM2.5 particles (particles that have a diameter of less than 2.5 μm) generated from the function of an FFF printer, using ABS filament material. The authors suggest that the generation and aggregation of the particles is manifested in four stages. The concentration increases at a slow rate at the first stage, and ultrafine particles are being generated. Subsequently, at the second stage, the concentration increase becomes more rapid. Afterwards, aggregation begins to take place, slowing down the increase of concentration (third stage), and finally, at the fourth stage, both concentration and aggregation sizes increase rapidly. Particles collected from the second stage were observed to be submicron in size (650 nm–1 μm), while micron-scale particles of increased volume were found during the fourth stage. Interestingly, humidity was found to be an influencing factor in the concentration and aggregation of the particles. Increase in relative humidity at a range of 40–80%, was found to result in an increase in captured particle size, and PM2.5 concentrations increased accordingly, while small particles were generated at an accelerated speed. The growth of micron-sized particles was found to be slow under the low humidity conditions. The effect of humidity was found to be greater during the fourth stage [93].

Gu et al. [94] used a desktop 3D printer with ABS filament material within a standardized 30 m^3^ environmental test chamber to study the effectiveness of control measures such as a filter cover and an air purifier with different filters in reducing exposure to UFP and VOCs. The filter cover was reported to provide the highest reduction in UFP exposure (93%), compared to lower reduction provided by the air filters (89–74%). This finding suggests that implementing control measures at the source location is more efficient than trying to remove particles after emission. Reducing VOCs was found to be a significantly more complex task, as it was observed that the use of the control devices results in the emission of several new VOCs [94].

In another recent study, Ding et al. [95] characterised the formation mechanisms of emissions from PLA, ABS and PVA filaments. The authors designed experiments using a combination of Evolved Gas Analysis (EGA) and Thermogravimetric Analysis (TGA) methods, aiming to closely simulate the heating process that takes place in an FFF 3D printer nozzle. It was demonstrated that emissions initiate at the start of the glass transition process and peak during liquefaction. All filaments started to generate VOC from about 75 °C to 150 °C, temperatures higher than their glass transition temperature and significantly lower that the temperature settings commonly used for printing. Furthermore, this study suggests control methods that may lead to reduced exposure. As experiments showed the vapour generation rate of a sample during the first heating cycle was evidently larger than subsequent reheated cycles, a phenomenon that the authors attributed to the release of “light components”, it is suggested that filament reuse could be an effective method for emission reduction. Low heating rates reportedly result in no styrene (which can display carcinogenic properties [96]) emissions from ABS filaments, in contrast to higher heating rates that lead to styrene emission peaks, reflecting the potential to utilize low heating rates to reduce the hazard of ABS emissions. This is also the first study in which emissions of UFP from the nozzle is directly observed and visualised through laser imaging. The minimum temperatures that the first fumes were observed were 120 °C, 178 °C and 185 °C for PVA, ABS, and PLA, respectively; these fumes are a mixture of nanoparticles and concentrated VOC emissions [95].

Davis et al. [97] studied FFF particle and VOC emissions and identified 216 individual VOCs that can be emitted in connection with FFF 3D printer processes, depending on the filament material. Five printers and twenty filament materials including ABS and PLA were tested and over 30 known or suspected irritants and carcinogens were detected for each filament material type. HIPS and ABS were found to display the highest TVOC emission rates, followed at a much lower level by PLA and PVA, while VOC emissions from nylon filaments displayed inconsistency between the two tests performed. ABS emitted the greatest amount of different VOC species, reaching 177 individual VOCs. A nylon filament and some ABS filaments were found to result in VOC emissions that exceeded the recommended limits for personal exposure. Several other parameters were shown to affect the magnitude of VOC emissions. Depending on printer brand, the results showed that between two tested printers, TVOC and particle emissions can be increased twofold for ABS and, to a lesser degree for PLA. Emissions from red ABS included more VOC species compared to green and white ABS, and the TVOC emissions were higher. The use of white PLA resulted in higher TVOC and particle emissions compared to black PLA filament. It is important to note that natural PLA displayed the lowest emission rates, and its particle mass emissions could not be quantified due to not exceeding the detection limit. It can be assumed that additives such as colour pigment have great influence on the emission potential. Filament brand was also reported to be highly important, affecting emission rates, the chemical identity of the emissions and the emitted particle size. It is important to note that emission of VOCs and particles were inversely related for the filament brands examined. Higher VOC emitting filaments displayed reduced releases of smaller particles for PLA and ABS alike. Consistently with other studies, increased nozzle temperature (from 230 to 255 °C) was found to lead to higher VOC and particle emissions, although affecting particle emissions more than VOCs [97].

Byrley et al. [98] accumulated and analysed data from the literature concerning 3D printer emissions and calculated 40 nm as the mean size of the particles emitted from 3D printing processes using PLA. The authors also determined that the mean PNC concentration according to the literature was 65,482 #/cm^3^ for PLA printing and 300,980 #/cm^3^ for ABS printing [98]. Interestingly, these concentrations both exceed the proposed 40,000 #/cm^3^ exposure limit that corresponds to biopersistent nanoparticles, with ABS presenting significantly higher concentrations than suggested to pose hazard.

In Table 1, a summary of crucial literature findings, relevant to the structuring of an FFF SbD scheme is demonstrated. A selection of suggestions for further research and proposed risk reduction measures found in the studies examined is also presented.

#### 2.4.3. Toxicity of Emitted Particles

Seeing that release of particles from 3D-printed processes has been confirmed under several different print configurations and workplace conditions, and exposure is reasonably expected, it is vitally important to note that the toxic characteristics of the emitted particles have been evaluated as well. Zhang et al. [99] have demonstrated that particles emitted from consumer level printer operation using PLA and ABS filament material can display comparable particle oxidative potentials to those of PM2.5. Quite interestingly, in vitro and in vivo investigations performed in this study showed toxic responses being manifested from both PLA and ABS-emitted particles. Moreover, even though PLA is considered a material of lower hazard, particles emitted from PLA processes brought about higher toxic response levels than ABS-emitted particles at comparable mass doses. PLA emitted particles were compositionally similar to the PLA monomer, while this similarity was not present in the ABS emitted particles. Additionally, PLA emitted particles were comparatively smaller (14 ± 25 nm average diameter) than their ABS counterparts (71 ± 20 and 106 ± 20 nm average diameter for each type of ABS tested), a characteristic that is highly likely to have determined their higher toxic potential [99]. Another study, performed by Farcasa et al. [100], confirms the in vitro toxic potential of ABS and Polycarbonate (PC) emitted particles. Emissions from a commercial 3D printer were collected, characterized and used for exposure of human small airway epithelial cells. Exposure to the emitted particles resulted in increased production of Reactive Oxygen Species (ROS), expression of pro-inflammatory cytokines and chemokines, and cell apoptosis and necrosis. This study also confirms presence of metal traces (Ni, Cr, and Fe) in particles released from ABS filament, while the authors suggest that this may be one of the causes of ROS production, and consequently, the display of toxic effects [100].

#### 2.4.4. Health Issues Connected with FFF 3D Printer Use

Chan et al. [101] conducted a small-scale survey of 46 occupational users of FFF 3D printers in 17 different companies, in order to identify potential correlations between 3D printing and the emergence of adverse health effects. 59% of participants reported displaying respiratory symptoms at least once per week in the previous year. The diagnosis of asthma or allergic rhinitis was substantially more likely for employees involved with 3D printers more than 40 h a week. It should be mentioned that more than half of the participants (52%) reported not using any form of personal protective equipment (PPE). The authors suggested that further studies should be undertaken to provide further understanding on the risks presented for workers [101]. Gümperlein et al. [102] investigated the acute effects of desktop 3D printer emissions on 26 human volunteers. Subjects were exposed to emissions during ABS (high UFP-emitter) and PLA (low UFP- emitter) printing, for 1 h, while several sensitive biochemical measures were analysed before and after the exposure. It was demonstrated that this exposure had no acute inflammatory effect in nasal secretions and urine. Indisposition and odour nuisance were reported to be increased for ABS exposure. The study took place in an exposure chamber of 32 m^3^ volume, used for occupational and environmental exposure assessment [102].

House et al. [103] reported a case study of a patient that developed asthma upon starting to work with multiple FFF 3D printers. Before being involved in 3D printing operations, the patient displayed no health issues, but had a history of asthma symptoms in childhood. The workplace where this individual was employed involved the operation of ten 3D printers in a room with a volume of 3000 cubic feet, and all printers used ABS filament. Adverse respiratory effects such as coughing, chest tightness and shortness of breath were observed 10 days after initiating printer operation. Five employees that worked in the same workspace did not experience such symptoms. Substitution of the ABS filaments to PLA filaments, reduction of the number of printers to five and use of an air purifier with a high-efficiency particulate air filter and organic cartridge improved his symptoms although still experiencing slight shortness of breath and chest tightness at work. His symptoms decreased over time after the exposure reduction modifications and eventually resolved [103].

#### 2.4.5. Emissions from Nanofiller-Containing Filaments

One of the benefits presented in lab-on-a-chip FFF 3D printing is the wide variety of available materials, which can be selected when distinct properties such as biocompatibility (PLA) or electrical conductivity (filaments with additives such as carbon black) are required. Commercial filaments that incorporate nanofiller materials such as carbon nanotubes [104,105] and graphene [106] are available, offering improved mechanical properties, or increased electrical conductivity. Utilization of nanoparticles as additives in the fabrication stage of microfluidic chips has been explored and implemented successfully for manufacturing methods other than FFF, for the development of conductive or magnetic properties [107]. In an FFF application, Duarte et al. [108] manufactured an inexpensive microfluidic device that measured the size of microdroplets based on contactless conductivity detection. The authors used a commercial FFF 3D printer and a combination of ABS and CNT-doped PLA filaments. The conductive properties of the CNT-enabled filament were utilized to print integrated sensing electrodes, while microfluidic channels were printed from ABS [108]. It can be reasonably expected that use of nano-enabled filaments will provide access to such new capabilities and increased functionality within FFF lab-on-a-chip manufacturing. Nevertheless, while nanoparticle-containing filaments seem very appealing in terms of widening the performance potential of lab-on-a-chip devices, their use can transform the outlook on process safety significantly, for a number of reasons that concern nanosafety aspects.

As a foremost issue, the nanoparticle release potential of nano-enabled filaments ought to be understood and examined. Results from a study conducted by Stefaniak et al. [109] demonstrate that FFF 3-D printing with CNT-containing filaments emitted CNT-containing polymer particles in the submicron to micron scale size range. In this study, emissions of commercially-available filaments such as ABS, PLA, and PC filaments that contained carbon nanotubes (CNTs) were investigated and studied in contrast with filaments of corresponding materials with no nanofiller content. Printing with these nano-enabled filaments resulted in the release of respirable size polymer particles that contained CNTs. It was estimated that 7.2% of these respirable particles could deposit in the alveolar region of the lung, if emissions were to be inhaled. Crucially, it was shown that the objects produced with the nano-enabled filaments displayed protruding CNTs on their surfaces, and the authors called attention to the possibility of CNTs being released as a consequence of abrasive processing or machining as post-processing [109]. It is therefore highly likely that emitted particles from FFF processes using filaments with nanofiller content will contain amounts of this nanofiller material.

Another study that investigated the influence of nanofiller on FFF process emissions was conducted by Potter et al. [110], reporting increased risks for such filament materials. The authors quantified and characterized VOC emissions of two commercial 3D printer filaments (ABS-CNT nanocomposite, ABS) in simulated FFF thermal conditions. In agreement with other studies [80,92], styrene was found to comprise the highest fraction of total VOC emissions. The authors report that printing a few hundred grams of filament can result in the 1 mg/m^3^ IRIS (Integrated Risk Information System) reference concentration for inhalation exposure for styrene being exceeded, depending on room size and ventilation. Interestingly, the presence of CNTs resulted in slightly reduced total VOC emissions under most experimental conditions, nevertheless increasing the emission of specific highly hazardous VOCs. It is also highlighted that emitted CNTs can present additional inhalation hazard as adsorption sites for VOCs [110].

Regarding nanotoxicity, as is to be expected, the toxic response to nanomaterials will be highly dependent on dose received. Safe levels of chemical exposures are commonly described by the corresponding Occupational Exposure Limits (OELs). To date, no occupational exposure limits dedicated to nanomaterials have been determined in EC legislation. Due to the increased surface area compared to the corresponding bulk material, as well as structural diversity often observed in nanomaterials, the Occupational Exposure Limit of the bulk material should not be considered directly relevant to the nanomaterial of the same chemical composition. It would be highly inaccurate to employ the OEL of carbon black (3.5 mg/m^3^) [111] to graphene or CNTs. The nanomaterial OEL is expected to be in a very lower range.

Exposure limits have been proposed by several organizations for some nanomaterials. Alternative risk assessment approaches involve calculated Benchmark Exposure limits. The British Standard Institute (BSI) [112] and The German Institute for Occupational Safety and Health of the German Social Accident Insurance (IFA) [113] have suggested methods for developing benchmark exposure limits for nanomaterials based on information such as density and OEL of the parent bulk materials. Based on these guidelines, the proposed limits for graphene are the following: BSI: 165 μg/m^3^, IFA: 40,000 #/cm^3^. The limits proposed for carbon nanotubes are significantly lower (1 μg/m^3^ by NIOSH [114]), as the biological behaviour of CNTs has been connected with asbestos fibres [115], the toxic effects of which have been described in detail [116]. Nevertheless, such exceptionally low OEL values indicate that utmost attention should be given in cases where carbonaceous nanoparticle exposure is expected. These resolutions suggest increased significance for the emission potential when using nanofiller filaments. An important consideration is that in many cases nanoparticle concentration will not be reported for the commercial filaments. Therefore, assessing safe levels of exposure will involve additional difficulties, rendering the adoption of a precautionary stance as vital.

## 3. Discussion

The studies examining FFF particle and VOC emissions do not always display absolutely consistent results, in terms of the magnitude and severity of the emissions, varying according to print conditions and materials employed. However, the emission of ultrafine particles and possibly, of some hazardous substances from 3D printers, in concentrations that may be cause for concern, has been demonstrated. The manifestation of toxic effects as a result of particle exposure is likely. In response to these findings, the design of the lab-on-a-chip manufacturing through FFF should be adjusted correspondingly. Additionally, lighter printed objects, short printing times and printing of small or complex objects may cause higher emissions [77,84]. Lab-on-a-chip devices fulfil these criteria, further highlighting the need for a SbD approach. In the following section, a Safe-by-Design strategy for FFF processes is presented, in line with the mitigation action hierarchy presented in Section 2.1: Structuring a Safe-by-Design Approach.

### 3.1. Hazard Minimization

#### 3.1.1. Material Selection

A basic SbD framework can be established with choosing a low-emitting filament material. In general, literature suggests that PLA filaments are safer, although hazard is not completely eliminated. Moreover, there seem to be several additional emission determinants that should be taken into account when choosing a printing filament, while required printed item properties may mandate the use of a specific material. It has been reported that knowledge about the precise chemical composition of the filaments, as well as the included additives is limited, and this information is confidential in many cases. Safety data sheet (SDS) documents contain limited information of the chemical identity of the additives used. These additives may display an important role in the characteristics of the emitted particles and substances [80,117]. New filament materials are continuously being developed, while the exact constituents of these products, and, consequently, their hazard levels are not known. Therefore, difficulties may be presented in assessing and comparing the safety levels of different filament products without performing a case-by-case investigation. A precautionary stance is suggested in case of limited information. Filament brand has been determined as an influencing parameter, and low emitter brands should be ideally selected, if such information is available. As additives and pigments are considered to be an important determinant of emissions, natural coloured polymers should be preferred if no special properties are required. The use of natural or transparent PLA as a low VOC emitter, as seen in [97], corresponds positively with the transparency requirements of lab on chip devices, suggesting natural/transparent PLA as a viable filament choice. Apart from reduced emission potential, the appropriateness of PLA is further enhanced by its biocompatibility [118].

#### 3.1.2. Equipment Selection

Printer brand has been demonstrated to influence emissions. Data on specific printer device emissions will most likely not be available, or will be difficult to assess, as other influencing factors will most probably be at play in any exposure assessment study as well. Employment of low emitting printers is preferred. Printer should be equipped with an enclosure if possible, in order to drastically reduce release into the working environment. Enclosures offer the additional benefit of reducing the possibility of contact with any hot surfaces or moving components. A heated print bed is a helpful feature, as this facilitates reliable adhesion of printed objects to the printing platform, while also reducing the need to use print adhesion aids such as sprays, that present possibilities of device contamination which could prove detrimental for biological applications. Capability for remote monitoring (e.g., through video recording) of print progress and potentially remote control of the printer can offer great protection from exposure. Selection of high-resolution printers for accurate microchannel construction is preferable. As layer height has been demonstrated to be one of the most influential factors for manufacturing efficient LOC devices [37,38], minimum layer height capabilities should be assessed, in accordance with the dimensional requirements for the printed devices.

#### 3.1.3. Fire and Explosion Hazard Minimization

Fine powders are confirmed to pose explosion risks, particularly organic and metallic powders. Particles emitted from FFF processes contain carbon and potentially metal traces [77,84,100]. Even though there seems to be no conclusive data on what explosive properties to expect from particles emitted from the FFF printing processes, a tendency for smaller particles to be susceptible to easy ignition is confirmed for the microscale [57] and may apply to the nanoscale, up to a certain threshold. Depending on the work practices and the control measures employed, confinement of ultrafine dust particles may occur, and the risk may be manifested.

Additionally, filament additives and exact chemical composition are generally not disclosed, which makes the prediction of emitted VOCs and particles challenging. If a flammable gas is simultaneously present along with combustible dust, it is considered that the explosibility is increased. Minimum explosive concentration, minimum ignition temperature, and minimum ignition energy are reduced, along with an increase in maximum rate of pressure [55]. Styrene has been observed as a prevalent substance in VOC emissions when printing using ABS filament material [69]. Regarding PLA, a main emitted VOC was found to be Methyl methacrylate [69]. These substances are characterised with a Level 3 NFPA Fire Rating [119,120]–Liquids and solids that can be ignited under the most ambient conditions [121]. Therefore, the most widely used filament types seem to present this potential for increased hazard. Other flammable VOCs that have been observed are ethylbenzene [67] and acrylonitrile [122]. Explosibility can be reasonably expected to be increased in such cases, to varying extents, depending on the removal capabilities from ventilation and the emission rates. Another factor that may influence explosibility are metal particles that have been reportedly been observed in 3D printing emissions. Zinc and Iron are susceptible to combustion [58] and have been detected in particle emissions [77]. The cumulative effect of the presence of flammable substances, increased particle concentration in confined spaces and small particle size, may lead to explosion hazards.

It is important to note that sprays used to promote adhesion, may increase flammability risk, and may present a fire risk as of themselves [60], as these substances can be highly flammable, and may be used frequently, if print completion time is fast and new prints are commenced continuously. Adhesion to build plate should be provided by adjustments in build plate temperature, so as to eliminate this hazard, and if sprays are to be used for these auxiliary purposes, adequate ventilation must be ensured.

Precautionary SbD measures can be the proper grounding of electrical equipment and operation of printers in adequate distance from hot surfaces and other heat producing equipment. Ignition sources should not be generated within the workspace. Smoking should not be allowed in 3D printing workspaces. Operation of multiple printers in the same workspace, and printing continuously for extended periods of time is expected to pose greater explosion risks, as the concentration of particles has been reported to increase, increasing the probability of exceeding the minimum explosion concentration threshold. Inherent safety modifications where particle and VOC generation is kept at a minimum also apply for reducing explosibility hazard. In general, exposure controls, like providing adequate ventilation, will most certainly diminish any fire risks to a great extent, as high amounts of airborne dust will accumulate only on very poorly ventilated workspaces.

It should be noted that a case where printer operation resulted in a fire bursting has been reported [123], although the cause of the fire is not known. The printer was left unattended while functioning and the damage caused was significant. Printers should not be left unattended for extended time frames.

### 3.2. Hazardous Emission Minimization–Adjustment of Print Settings

Adjustment of print parameters ought to be in line with LOC manufacturing quality requirements and process impediments, but also take into consideration emission minimization. Examination of the literature findings on emission determinants can guide on print parameter optimization for both emission reduction and functionality.

#### 3.2.1. Shell Number and Layer Height

Optimizing the size of the outer wall of the print structures has been demonstrated to produce builds of increased leak integrity. Studies have shown that four shells are adequate to reduce the potential for leaks [40,41]. Additionally, small layer heights (e.g., 60 μm [37]) have been observed to produce structures of increased transparency, so it is reasonable to suggest this setting to print microfluidic devices. Through this approach, increased mechanical stability may also be achieved [124]. These settings are not expected to significantly influence emissions as of their own, nevertheless increasing print time and, thus, the timeframe in which emissions can occur.

#### 3.2.2. Temperature

It is acknowledged that higher temperature settings on both extruder and build plate correspond to higher emission potential. As seen in the literature, successful PLA microfluidic device manufacturing has been demonstrated using relatively high temperature settings. Extruder temperatures were reported to be adjusted at 215–230 °C and bed temperatures to 60–70 °C [38,40], values that are considered to be comparatively high for PLA. In order to mitigate release, utilization of the minimum applicable temperature is suggested. PLA prints have been reported to display homogenous quality and acceptable deviations when temperature is reduced within functional range [71], although this may not be the case for the more demanding in terms of dimensional tolerance lab-on-a-chip manufacturing. Lower temperature has been demonstrated to result in leak issues in devices printed with ABS where an extruder temperature of 230 °C was used as opposed to achieving successful leak-free characteristics with 240 °C and other parameter optimization [41]. In cases where temperature reduction is totally not applicable, the focus should be placed on implementing release and exposure mitigation measures by other means. However, higher temperatures than those suggested by the filament manufacturer should not be used, both for ensuring proper print operation and preventing malfunctions, as well as preventing increased emissions resulting from inappropriately high temperature [71].

#### 3.2.3. Infill

Infill can also be adjusted to reduce emissions, although definition of this setting is most likely to be governed by the requirements for mechanical integrity or functionality of the lab-on-a chip device. The successful manufacture and testing of such devices with 100% infill density has been reported [35,38], and increases in infill density have been demonstrated to diminish the potential for leakage [39]. However, infill adjustments other than 100% may be required in order to keep cost within a certain low range. Moreover, along with an increase of infill, print time is also increased, leading to an overall lengthier printing process, affecting the timeframe within emissions occur accordingly. As demonstrated in [73], higher infill is expected to beneficially affect emission potential within a low infill density range (10–30%), reducing peak emission values. Additionally, lower emitting infill patterns such as linear infill can be utilized, at the expense of achieved strength, while additionally, for reduction of the peak emission magnitude resulting from the bridging layer, the 1st top layer can be adjusted to display a lower feed rate, as seen in [73]. This is a feature that is present in advanced slicer software and may be useful in reducing emissions.

#### 3.2.4. Print Speed, Feed Rate

Although print time is expected not to be very high for LOC devices which display shape complexity but small dimensions, long printing sequences can be reasonably expected, as continuous prototyping or production will be sought. Comparatively slower print speeds have been suggested to beneficially affect printing quality for LOC and specific extrusion speed settings that have been successfully employed include 30 mm/s [38], 40 mm/s [40], 60 mm/s [41]. Reduced print speed is therefore considered useful for this specific application. Slow feed rate (30 mm/s) [71] has been reported to be an appropriate setting for reducing emissions.

### 3.3. Exposure Minimization

#### Ventilation

A well-ventilated area should be used for FFF 3D printing. A local ventilation system with High-efficiency particulate air (HEPA) and carbon filtration, dedicated to the printer, can be implemented for more efficient protection from emissions. Proper ventilation practices for laboratories using potentially hazardous materials can be applied for 3D printing workspaces, and involve ensuring a minimum of 6 air changes per hour [125]. The minimum required air change rates should be scaled upward based on increases in anticipated usage and number of printers employed. Installing no more than one printer per standard office space (45 m^2^) for typical office conditions is suggested. It has been proposed to maintain a minimal negative air pressure differential with respect to adjacent spaces for the workspace where printer operations take place [84]. This is achieved when exhaust air outflow is slightly more than the room supply air volume. A general guide provided by the American Conference of Governmental Industrial Hygienists (ACGIH) suggests a 5% flow difference between supply and exhaust air flow rates but no less than 0.85 m^3^/min, although precise setup will depend on the specific conditions of the work area [126].

### 3.4. Protection from Exposure

#### 3.4.1. General Administrative Practices

- Operation of 3D printers should only be allowed for trained and authorized employees. Untrained personnel should not use the devices, unless under the supervision of an authorized employee.
- Rules and standard operating procedures for each printer and print configuration should be readily available for employees.
- Safety Data Sheet documents ought to be available and accessible for all print media and chemical substances used in the context of the printing.
- Proper hygiene measures corresponding to general laboratory processes can be applied to 3D printing as well. Food and beverages should not be allowed in the 3D printing operation locations. After 3D printing work, employees should wash hands thoroughly.
- Cleaning of work surfaces where particle deposition is expected should take place periodically. Wet cleaning methods should be preferably employed, in order to prevent resuspension of particles and secondary exposure episodes.
- Covers and enclosures should not be opened while printer is operating, unless there is a specific need for interaction with the print or the components.
- Waste materials such as failed prints and support structures can be disposed of and treated as normal waste. Sharps should be disposed in a sharps bin. Nano-enabled filaments may contain toxic materials and may constitute toxicity hazards, necessitating special care in waste handling.
- Employee presence in close proximity to the printer should be minimized if possible. If enclosures are employed, presence of employees at the time of the enclosing hood opening should be minimized. A crucial timeframe for emissions is considered to be the precise initiation of the printing process, often reported to display the maximum UFP number concentrations out of the entire printing process [65,69,74]. This should be taken into account, in an effort to minimize employee presence during this high emission potential stage.
- Printer(s) should preferably be placed in a room dedicated to printing processes, and not in areas shared with office workspaces. As demonstrated in [64], there may be complexity to be observed in the relationship between particle concentration and distance from printers, as a consequence of coagulation, while emitted substances and particles can remain in the breathing atmosphere for long after print completion [77], if not removed by the exposure control systems. Maintaining distance from printers, especially for extended periods of printer operation can be a functional practice for exposure reduction, but may not eliminate the risk. A risk control methodology should not be solely based on ensuring employee distance from equipment, but controlling emissions and providing adequate ventilation.
- As a means for cultivating a constructive 3D printer safety outlook, near misses and potentially hazardous incidents during printer use should be documented.
- The cleaning and maintenance tasks involved in long term employment of 3D printing processes can be structured around safety guidelines as well. Cleaning and maintenance staff should be acquainted with the 3D printing instruments, assessing cooling time after use, interior build space, integrity of mechanical components and moving and heated parts when working, in order to avoid unwanted interference with proper function or potential injuries.
- Harsh chemicals and strong organic solvents for cleaning purposes should be avoided, as they may interact with other materials. Alcohol based wet wipes are considered suitable. For cleaning, disposable paper towel should be used.
- The exposure control system should be regularly maintained, in terms of replacing enclosures, and replacing filters when necessary.

#### 3.4.2. Protection from Printer Operation-Related Hazards

- Clear signage should be installed to indicate risk from hot surfaces, moving parts and potentially hazardous airborne agent concentrations.
- Proper laboratory practices for safe processes with moving machinery involve not interfering with the moving parts of the machinery during function. Loose clothing and jewellery can be entangled in the moving parts and should be avoided.
- Hot surfaces should not be interacted with while printer is operating or during the preheating stage, and hand protection should be used during necessary interaction with the hot parts. In case of contact with hot surfaces, burns should be treated with cold running water as soon as possible. Sufficient time should be allocated for the print and the heated printer components to cool down after print completion, before any direct contact.
- Regular scheduled printer maintenance procedures and electrical inspection tests will be adequate to monitor and treat any electrical hazards.
- Post processing procedures with solvents have been demonstrated to display potential for VOC exposure [91]. Therefore, if they need to be applied, they should be performed in well ventilated locations. Additionally, ventilation of the general printing area should be ensured during bed preparation procedures such as spray use, if such operations are needed.

#### 3.4.3. Administrative Practices Related to Specific Operations

- If ventilated enclosures are used, sufficient time should be allocated after print completion for printer emissions to clear (i.e., the hood clearance time) before opening the enclosing hood. This time interval could be approximately 20 min [89], and can be reduced by increasing the airflow rate and/or reducing the volume of the enclosure. As emitted particle decay or deposition in surfaces is expected to occur [91], it would be reasonable to apply this practice in case simple enclosures are used as well, although the time allowed for particles to settle could be substantially higher.
- In connection with [93] where increased humidity has been proven to enhance particle size growth, adjustment of humidity within the printing workspace may be an approach to promoting the growth of emitted particles to less hazardous larger agglomerates, although sustaining high humidity may present practical issues. 3D printing filament printability and print operation can be adversely affected by the presence of moisture in the air [127]. This could be a low priority measure, employed in cases where multiple printers need to be operated and further risk mitigation is required.
- When jams occur, upsurges in release are expected, and a waiting time for ventilation before opening enclosures has been suggested [74]. For extended protection, a prevention-through-design modification that involves an automated shutoff for the nozzle heater in the event of a jam has been proposed [74]. Print pauses have also been found to induce emission upsurges [76], as well as malfunctions [86], so that should also be taken into account when responding to such episodes.
- Air purifiers have been successfully implemented to diminish ultrafine particle exposure from 3D printers [94,103]. If additional exposure reducing measures are required, installation of air filtration systems can be useful. It is important to note that the system selected for this purpose ought to have both carbon and high-efficiency particulate air filtration capabilities.
- Clearance and cleaning of the nozzle can attenuate emission peaks [76], and should be performed frequently, as it can also contribute in malfunction and blockage/clogging prevention. This operation ought to be undertaken using appropriate protective equipment.
- Optimization of the printing procedure so as to eliminate filament heating may be used as an emission reducing measure, as seen in [71].
- If use of filament materials or process parameters that present increased hazard (like ABS or nano-enabled filaments) is required, these specific processes can be scheduled for less populated working hours, where reduced personnel exposure is reasonably expected.
- Prolonged use of printers may lead to increased emission potential [77]. This is an additional factor that needs to be considered when assessing risks in long-term 3D printer application, necessitating the systematic revision and update of exposure assessments in such cases.
- If installation of a full enclosure is absolutely not applicable, employment of a partial enclosure may provide a minimum level of protection [69,74].
- Individuals diagnosed with asthma or other respiratory health issues, as well as individuals with any history of respiratory issues should use these instruments with increased caution, as they may be more susceptible to display adverse health symptoms in response to the emissions [103].

#### 3.4.4. Personal Protective Equipment

- Respiratory protective equipment should be employed as a last priority measure, if emission and exposure control are absolutely not applicable.
- Gloves should be used when interfering with hot surfaces. Handling of prints made of nano-enabled filaments with gloves is also recommended, as the presence of nanoparticles on such object surfaces has been confirmed [109].
- Eye protection should be used during post processing activities, as certain procedures such as removing supports or additional print structures with sharp objects may lead to projectiles and flying debris. Employees involved with such Post processing activities should be equipped with gloves and lab coats as well.
- Any PPE recommendations displayed in the SDS documents should be adhered to.

## 4. Conclusions

**Figure 6:**
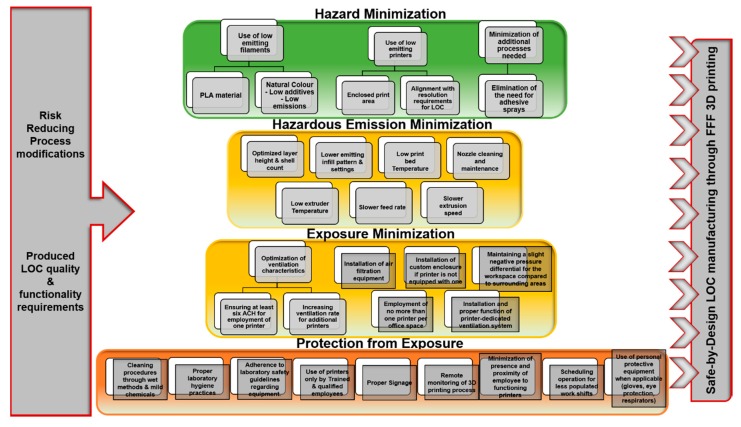
Summary of the SbD strategy for LOC manufacturing through FFF 3D printing.

Figure 6 summarizes the outcome of the work presented in this study, as a synopsis of the proposed SbD measures to be implemented in the context of LOC fabrication through FFF. Identified risk reduction measures have been cross-evaluated with requirements regarding LOC quality and functionality in terms of compatibility and are presented as practical guidelines that can be employed. Further research on the emission magnitude determinants of FFF 3D printing will facilitate greater precision and confidence in establishing safe FFF processes. Implementation of the risk mitigation schemes and evaluation of their performance will allow further optimization of the SbD strategy.

## Figures and Tables

**Table 1:** Synopsis of research findings regarding emissions and risk mitigation measures in the literature examined.

Reference	Filament Materials Tested	Main Highlight Findings Regarding Emissions	Suggestions and Identified Risk Mitigation Measures
Stephens et al. [63]	ABS, PLA	Both materials are high emitters. ABS displayed higher emission rates than PLA. Emitted particles were solely in the nano-scale range (< 116 nm). ABS-emitted particles were smaller compared to particles emitted from PLA.	Installation of ventilation and air-filtration systems.
Zhou et al. [64]	ABS (two colours)	Higher particle concentrations were observed for positions further away from the printer. No ultrafine particle data reported. Simultaneous operation of two printers led to increased concentrations.	Indoor ventilation for removal of particle contaminants.
Kim et al. [65]	ABS, PLA (two types)	Higher emissions reported for ABS. One type of PLA emitted mostly non-ultrafine particles. High upsurges in ultrafine PNC were observed during print operation initiation. Emission of hazardous VOCs (e.g., formaldehyde) is possible.	Use of safer filament materials, use of filters/adsorbents.
Azimi et al. [69]	ABS, PLA, HIPS, nylon, laybrick, laywood, polycarbonate, PCTPE, TGlase	ABS displayed the highest emission rates, while PLA displayed the lowest. High print bed temperatures were connected to high emission rates. Extruder temperature presented reduced significance in determining emissions. A partial enclosure can diminish the exposure potential to a minimum extent.	Development of low-emitting printing materials and technologies. Use of sealed enclosures for devices. Use of filtration systems.
Deng et al. [71]	ABS, PLA	ABS produced higher emissions than PLA. Middle range feed rates resulted in higher emissions compared to slower or faster feed rates. The heating stage is reported as the most crucial for emissions. Increase of nozzle temperature leads to increased emissions. When using temperatures high enough to approximate decomposition temperature, emissions can rise significantly.	External pre-heating before filament loading drastically reduced emissions. Development of optimized printer cooling systems in relation to particle emissions.
Cheng et al. [73]	ABS	Use of the hexagonal infill pattern resulted in higher emissions compared to other patterns. Higher infill density reduced emissions. The “bridging” effect results in high emission peaks.	Use of “linear” infill pattern and higher infill density. Slower feed rate in the 1st top print layer can reduce emission peaks.
Yi et al. [74]	ABS, PLA (4 different colours for each material)	PLA emitted particles were smaller compared to ABS. Filament colour influences emission concentrations and emitted particle size. Nozzle jams can induce emission peaks. Loose covers can offer moderate protection from exposure.	Use of enclosures, use of printers in a well-ventilated space, direct ventilation of the printer, maintaining distance from the printer. Selection of safer materials and printer devices. Optimization of response to nozzle jams to reduce exposure.
Zhang et al. [75]	ABS, PLA, Nylon	Most particles emitted were in the ultrafine size range. Influencing factors for emission magnitude were extruder temperature, filament brand, filament colour and build plate temperature.	Development of standardized testing and data analysis methods. Toxicity evaluation of emitted particles.
Simon et al. [76]	ABS	High emission peaks just before the print initiation stage, and smaller peaks during print pauses were observed. For particles larger than 420 nm, no statistical difference in PNC was observed, while most emitted particles were less than 25 nm in diameter.	Nozzle cleaning and clearance can result in emission reduction.
Steinle [77]	PLA, ABS	Prolonged use of printers can lead to higher emission rates. Lighter objects and objects requiring short print duration can display higher emission rates. Printing in a large and well-ventilated office is regarded as safe, as UFP and VOC concentrations were not significantly increased. In areas with poor ventilation, contaminants can be detected for many hours after printing.	Hazardous levels of exposure are not expected for adequately ventilated workspaces.
Wojtyła et al. [79]	ABS, PLA, PET, Nylon	Although temperatures employed in FFF are lower than polymer decomposition temperatures, they can cause partial decomposition of polymers, inducing emission of volatile organic compounds. ABS displays higher hazard compared to PLA, presenting increased concentration of produced organic pollutants.	Printing in a well-ventilated workplace is considered safe, but continuous printing operations in poorly-ventilated areas may result in increased VOC concentrations. Development of dedicated 3D printing filters and exposure protection systems.
Wojtyła et al. [80]	ABS (various colours), HIPS, ABS + PC, Nylon, PET-g + carbon fibres, Polyamide + carbon nanotubes, nylon + carbon nanotubes, PLA (various colours)	Apart from polymer thermal degradation, thermal degradation of additives is a contributing factor for VOC emissions. Temperature, filament type and colour were found to be influencing parameters as well.	Development of new and advanced air filtration technologies.
Stefaniak et al. [83]	ABS (various colours), PLA (various colours), ABS+, Silver ink	Emission magnitude was determined by instrument design, filament material and print operation parameters. For the conditions studied, personal exposures to metals and organic chemicals were all below Recommended Exposure Limits.	Effective contaminant concentration reduction through a custom-built ventilated enclosure was confirmed.
Zontek et al. [84]	PLA, ABS	Higher printing temperatures resulted in higher number particle concentrations. In appropriate ventilation conditions, particle concentration is reduced in areas further away from the printer. In spaces with poor ventilation, continuous function of the printer can result in elevated concentration levels throughout the printing room.	Development of ventilation recommendations, determination of suitable printer locations. Operation of printers without enclosures only in large, highly ventilated spaces. Maintaining the printer enclosure under slight negative pressure with respect to the surrounding areas.
Floyd et at. [85]	ABS, PLA, PVA, HIPS, PCABS, nylon, bronze- PLA, and PET	Highest particle emission potential was displayed by ABS+PCABS, bronze-infilled PLA, and PVA. Particles emitted presented a modal size of less than 100 nm, and also rod-shaped fragments were observed. These dimensional qualities lead to higher probability of penetration to the alveolar region of the respiratory tract.	Personal exposure assessment through field studies. Performance of toxicological studies. Investigation of the emission potential of post-printing processes.
Mendes et al. [86]	ABS, PLA	Increasing nozzle temperature led to increased emissions. Emission of very small (1–3 nm) nanoparticles was observed. Printer malfunction episodes can lead to emission peaks. Emission concentrations from PLA filaments do not exceed the representative exposure limit corresponding to biopersistent nanomaterials, but exposures from ABS filaments slightly exceed it. VOC concentrations detected were not expected to pose health risks.	Caution is suggested during simultaneous operation of multiple printers. Cleaning of the heated parts of the printer. Use of low extruder temperatures. Use of enclosures, local exhaust ventilation, and air filtering systems.
Health and Safety Executive [88]	PLA, ABS, HIPS, NinjaFlex^®^	All print operations emitted sub-micron particles. Increase of the nozzle temperature resulted in decreased emitted average particle size and higher emissions. PLA filaments were found to display reduced emissions than ABS, and ABS emitted particles were smaller than PLA. When testing identical filament materials, emission rates and emitted particle size varied for different printers.	Conducting more experiments under workplace conditions, for representative exposure assessment. Choosing low emitting filaments. Employing lower nozzle temperatures. Enclosing the printer and ensuring air filtration. Allocation of time for emission clearance before opening the enclosure.
Du Preez et al. [91]	ABS (various colours), PLA (various colours), PC, ultem	Particle emissions displayed variance depending on filament colour. Removal of printer cover results in particle emission peaks. TVOC concentrations increased during post processing activities.	Checking on prints without cover removal, through transparent viewing ports. Installation of filtration systems. Use of fume hoods when conducting post processing tasks.
Youn et al. [92]	PLA	Smaller particles were primarily emitted (dominant peak at 10 nm) during the initiation of printer operation while larger particles were emitted (dominant peak at 88 nm) after a short time interval. Low concentrations of several HAPs were observed.	Performing closed chamber studies to evaluate emissions. Quantifying dangerous substance doses in the human respiratory tract, towards producing complete exposure assessments.
Rao et al. [93]	ABS	Identification and analysis of the mechanisms and stages of emissions. Increase in humidity can lead to enhanced emitted particle growth.	Confirmed effectiveness of nanofiber-based membranes to capture particles emitted from 3D printing.
Gu et al. [94]	ABS	Most emitted particles were in the ultrafine size range. VOC emission from the exposure control devices employed was also reported.	A filter cover provided effective removal of UFP. Lower effectiveness in decreasing exposure was provided by an air purifier.
Ding et al. [95]	ABS, PLA, PVA, Nylon	Emissions of UFP from the nozzle were directly visualised. Emissions initiate at the start of the glass transition process and peak during liquefaction of the filament.	Filament reuse is suggested as a method for emission reduction. Low heating rate can restrain the formation of hazardous substances.
Davis et al. [97]	ABS, PLA, Nylon, PVA, HIPS (several different types)	Filament colour was found to be a significant influencing factor for VOC emissions. Natural coloured filaments were reported to lead to very low VOC emissions. HIPS and ABS displayed the highest TVOC emission rates. ABS filaments emitted numerous different VOC species. Both VOC and particle emissions increased with higher extrusion temperature. Filament brand was also an important determinant, with filaments displaying high VOC emissions releasing fewer and smaller particles for both ABS and PLA.	Use of low-emitting filaments. Decrease of the printer nozzle temperature. Providing adequate ventilation.
